# Complete genome sequence of the fire blight pathogen *Erwinia pyrifoliae *DSM 12163^T ^and comparative genomic insights into plant pathogenicity

**DOI:** 10.1186/1471-2164-11-2

**Published:** 2010-01-04

**Authors:** Theo HM Smits, Sebastian Jaenicke, Fabio Rezzonico, Tim Kamber, Alexander Goesmann, Jürg E Frey, Brion Duffy

**Affiliations:** 1 Agroscope Changins-Wädenswil ACW, Swiss National Competence Center for Fire Blight, Division of Plant Protection, CH-8820 Wädenswil, Switzerland; 2 CeBiTec, University of Bielefeld, Bielefeld, Germany

## Abstract

**Background:**

*Erwinia pyrifoliae *is a newly described necrotrophic pathogen, which causes fire blight on Asian (Nashi) pear and is geographically restricted to Eastern Asia. Relatively little is known about its genetics compared to the closely related main fire blight pathogen *E. amylovora*.

**Results:**

The genome of the type strain of *E. pyrifoliae *strain DSM 12163^T^, was sequenced using both 454 and Solexa pyrosequencing and annotated. The genome contains a circular chromosome of 4.026 Mb and four small plasmids. Based on their respective role in virulence in *E. amylovora *or related organisms, we identified several putative virulence factors, including type III and type VI secretion systems and their effectors, flagellar genes, sorbitol metabolism, iron uptake determinants, and quorum-sensing components. A deletion in the *rpoS *gene covering the most conserved region of the protein was identified which may contribute to the difference in virulence/host-range compared to *E. amylovora*. Comparative genomics with the pome fruit epiphyte *Erwinia tasmaniensis *Et1/99 showed that both species are overall highly similar, although specific differences were identified, for example the presence of some phage gene-containing regions and a high number of putative genomic islands containing transposases in the *E. pyrifoliae *DSM 12163^T ^genome.

**Conclusions:**

The *E. pyrifoliae *genome is an important addition to the published genome of *E. tasmaniensis *and the unfinished genome of *E. amylovora *providing a foundation for re-sequencing additional strains that may shed light on the evolution of the host-range and virulence/pathogenicity of this important group of plant-associated bacteria.

## Background

Fire blight caused by the enterobacterium *Erwinia amylovora*, a quarantine pathogen in Europe, is the most important global threat to pome fruit production (i.e., apple, pear) and to a wide-variety of *Rosaceae *(Maloideae), including amenity and forest species [1]. *E. amylovora *is native to the North Eastern USA, and it was the first phytopathogenic bacterium described (see [2]). This invasive pathogen was first introduced into the UK in the late 1950s and has spread across Europe over the past three decades, with continuing advance eastward that threatens the native origin of apple in Central Asia [3]. Fire blight symptoms include recurvature of shoots (shepherd's crook), necrosis, ooze, and cankers. The pathogen can enter through nectaries, hydathodes and wounds resulting in blossom, shoot or rootstock blight syndromes [4]. *E. amylovora *growth is highly dependent on weather conditions, which has been used to develop disease forecasting models, and it is passively vectored by flower-foraging insects [5]. Epidemics can develop rapidly and result in death of individual plants or entire orchards within a single season, leading to severe economic losses [2]. *E. amylovora *has poor ecological fitness away from host or surrogate plants [4].

*Erwinia pyrifoliae *is a newly described pathogen, closely related to the main fire blight pathogen *E. amylovora*. *E. pyrifoliae *is primarily a pathogen of Asian or Nashi pear (*Pyrus pyrifolia*) and is considered to have a restricted geographic distribution in East Asia (Korea and Japan) [6-8]. Monitoring to detect *E. pyrifoliae *is rarely conducted, although a quantitative PCR method based on differential 16S rRNA and 16S-23S ITS sequences [9,10] has recently been developed; thus the precise distribution of this pathogen is somewhat uncertain. *E. pyrifoliae *causes fire blight disease symptoms essentially indistinguishable from those of *E. amylovora *infection. Limited host germplasm screening with *E. pyrifoliae *strains indicates a slightly wider host-range than originally thought, with virulence observed on selected commercial pear (*Pyrus communis*) and apple (*Malus domestica*, 'Idared') varieties [9]. The level of virulence on non-Asian pear hosts is markedly lower than that typically observed in *E. amylovora*, suggesting that important genetic differences remain to be elucidated.

In contrast to the considerable genetic information available for *E. amylovora *[11,12], relatively little is known about the genetic basis of virulence and environmental fitness in *E. pyrifoliae *[13]. As for *E. amylovora*, *E. pyrifoliae *intra-species genotypic diversity appears low, with strain differences primarily observed in plasmid content [14]. Thus far, only minor genetic differences have been found between *E. pyrifoliae *and *E. amylovora *[13,15].

The objectives of our work were to sequence the complete genome of the type strain of *E. pyrifoliae*, strain DSM 12163^T^, and then compare it to the recently sequenced genome of the non-pathogenic pome fruit epiphyte, *E. tasmaniensis *Et1/99 [16]. Identifying differential sequences among these related bacteria may provide useful insights into host-pathogen interactions that could eventually be exploited for fire blight control.

## Results and discussion

### General genome description

**Figure 1:**
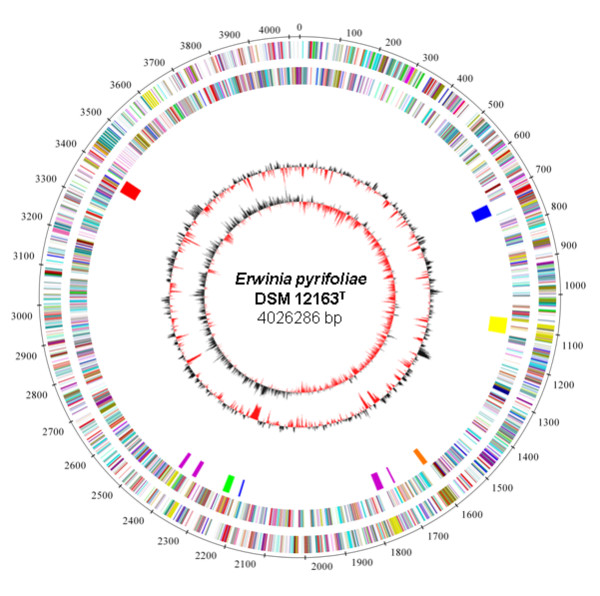
**Circular representation of the chromosome of *E. pyrifoliae *DSM 12163^T^**. Circles (from outside to inside) First: scale bar in kb; second and third: predicted coding sequences of *E. pyrifoliae *DSM 12163^T ^chromosome on the leading and lagging strand, respectively (colors according to COGs); fourth: coding sequences of the *hrp*/*hrc *T3SS (red), the *inv*/*spa *T3SS (green), the T6SS clusters (blue), the flagellar genomic island (yellow), dispersed flagellar gene clusters (purple) and the EPS biosynthetic cluster (orange); fifth, G+C content; sixth, G+C skew.

The genome of the type strain of *E. pyrifoliae*, strain DSM 12163^T ^(also archived as Ep16/96^T^, CFBP 4172^T^, and CIP 106111^T ^[7]), was sequenced using 454 pyrosequencing and Solexa sequencing, and assembled. The genome contains a circular chromosome of 4,026,286 bp (Fig. 1), and four plasmids of 35,901 bp (pEP36), 4,960 bp (pEP5), 3,070 bp (pEP3), and 2,610 bp (pEP2.6) (Table 1).

**Table 1:** General features of the genome of *E. pyrifoliae *DSM 12163^T^.

	Chromosome	pEP36	pEP5	pEP3	pEP2.6
Size (bp)	4,026,286	35,901	4,960	3,070	2,610
Sequencing coverage (454)	25.2	129.1	152.2	457.1	518.1
Sequencing coverage (Solexa)	34.9	179.2	214.5	648.2	710.4
Calculated copy number/cell	1	5.1	6.1	18.4	20.5
G+C content	53.41%	49.86%	53.12%	48.96%	44.58%
Coding sequences	3,986	40	7	4	1
rRNA operons	7				
tRNAs	74				

Annotation was completed using the genome annotation system GenDB [17], and manually optimized. In total, 4,038 open reading frames (ORFs) were predicted. The chromosome contains seven rRNA operons, and 74 tRNAs, which falls within the range observed for most free-living enterobacterial genomes. Several low G+C regions indicated the presence of horizontally acquired sequences. Inspection of some of these indicated the presence of several prophages in the chromosome.

### Plasmids

Earlier reports identified four plasmids in a large group of *E. pyrifoliae *strains, including strain DSM 12163^T ^[18,19]. Recently, the complete sequences of plasmids pEP36 and pEP2.6 from *E. pyrifoliae *Ep1/96 were published [8]. These two plasmids were found to be present in the genome of *E. pyrifoliae *DSM 12163^T ^with slightly differing sizes (35901 bp and 2610 bp, respectively). We sequenced and annotated two additional small plasmids of sizes 4,960 bp (pEP5) and 3,070 bp (pEP3).

From the coverage of the individual plasmids, it is possible to estimate the copy number of each plasmid in *E. pyrifoliae *DSM 12163^T ^(Table 1). According to this calculation, plasmid pEP36 and pEP5 are present in 5-6 copies per cell, while the small plasmids pEP3 and pEP2.6 have a medium copy number ranging from 18-20 copies per cell. This explains the high yield of plasmids reported in earlier studies, whereas it is more difficult to obtain plasmid pEA29 from *E. amylovora *present at approximately two copies per cell (T.H.M. Smits and B. Duffy, unpublished data).

**Figure 2:**
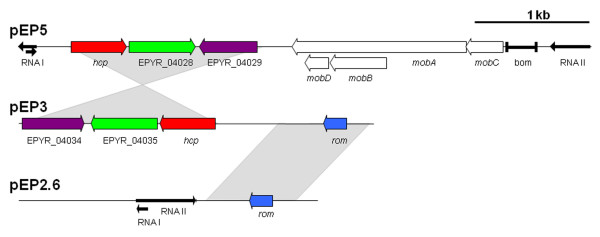
**Comparison of the annotated sequences of *E. pyrifoliae *DSM 12163^T ^plasmids pEP5, pEP3 and pEP2.6**. Genes with high homology are indicated in the same color. Grey shading indicate regions with DNA sequence identity.

Plasmid pEP3 shares a region (Fig. 2) with that of ColE1-type plasmid pEP2.6 [8], having 99.2% sequence identity over approximately 800 bp including the RNA I modulator gene but not covering the *oriV *region. The rest of the plasmid contains a large region that is similar (78% identity) to a part of plasmid pEP5 (see Fig. 2). The latter plasmid contains *mobCABD *genes similar to those on the ColE1-type plasmid pSW100 found in the phytopathogenic bacterium *Pantoea stewartii *subsp. *stewartii *SW2 [20], indicating that it may be a transmissible element. The region that is shared between pEP3 and pEP5 encodes three ORFs (see Fig. 2), two of which are hypothetical proteins and one which encodes Hcp, a putative type VI secretion system (T6SS) effector protein (see below).

### *rpoS *RNA polymerase sigma factor deletion

During genome assembly, it was observed that *E. pyrifoliae *DSM 12163^T ^has a 140 bp deletion in the *rpoS *gene (EPYR_03050), when compared to the *E. amylovora *OT1 or *E. tasmaniensis *Et1/99 *rpoS *gene sequence. This 140 bp deletion results in a frameshift and a coding interruption after the RpoS subregion 1.2 [21]. After this deletion, the remaining sequence of the *rpoS *gene resumes in a different frame, starting from subregion 2.4 in RpoS [21]. This deletion thus encompasses a highly conserved region that putatively interacts with the core RNA polymerase.

**Figure 3:**
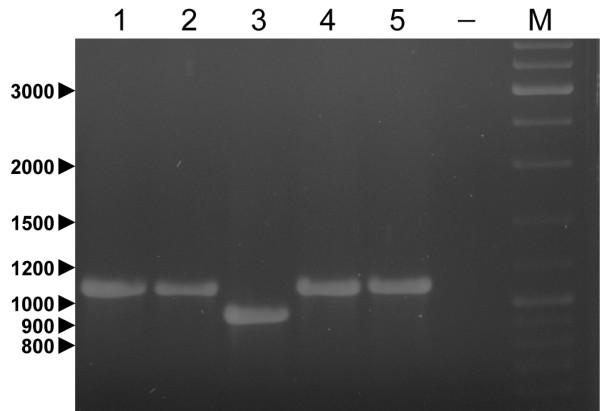
**Electropherogram of amplified *rpoS *genes from different *Erwinia *species**. Lane 1: *E. amylovora *CFBP 1232^T^; lane 2: *E. amylovora *OR29; lane 3: *E. pyrifoliae *DSM 12163^T^; lane 4: *E. pyrifoliae *CFBP 4174 and lane 5: *E. billingae *LMG 2613^T^. A minus sign denotes the negative control (-); M denotes the marker (Fermentas GeneRuler DNA Ladder Mix). Relevant marker sizes (in bp) are indicated at the left side of the figure. The *rpoS *gene was amplified using primer set Ep-rpoS-F (5'-AGTACTGGCACGAGTTCTGTTAGA-3') and Ep-rpoS-R (5'-TGCAGTATTTCACGCAGACGACGC-3'). The expected amplicon size of an intact *rpoS *gene is 1109 bp; for the 140 bp deletion the amplicon is 969 bp.

Using PCR primers designed in the *rpoS *region, we confirmed that this deletion is present in *E. pyrifoliae *DSM 12163^T ^(Fig. 3). However, the *rpoS *gene of *E. pyrifoliae *CFBP 4174 has the same size as the amplicon for *E. amylovora *strains CFBP 1232^T ^and OR29 and *Erwinia billingae *LMG 2613^T^, indicating that the deletion is specific for *E. pyrifoliae *DSM 12163^T^. This mutation may have occurred prior to submission to the culture collection, or represents an environmental adaptation in this strain, possibly associated with host-pathogen interactions or ecological fitness [22-24].

### Type III secretion system operons

Type III secretion systems (T3SSs) are widely distributed among proteobacterial pathogens of plants, animals, and humans, and constitute a fundamental virulence determinant [25]. In other bacteria, T3SSs were found to be critical for the establishment of non-pathogenic host-relationships with plants [26,27] and insects [28]. Mutations or absence of T3SS genes interfere or abolish bacterial-host interaction [29]. Generally, genes encoding elements of the T3SS machinery are conserved among bacterial species, but each system includes specific effectors targeting respective hosts. Proteins secreted by the Hrp complex of *E. amylovora *and other plant pathogenic bacteria are required for virulence on susceptible host plants and for the elicitation of a hypersensitive response in resistant or non-host plants [25,30].

The genome of *E. pyrifoliae *DSM 12163^T ^contains two distinct T3SSs. One T3SS is closely related to the *hrp*/*dsp *cluster of *E. amylovora*, while the second T3SS is more similar to the *inv*/*spa *cluster of *Salmonella typhimurium *[31]. The hypersensitive response and pathogenicity related region (*hrp*) of *E. pyrifoliae *DSM 12163^T ^is composed of 25 genes (EPYR_03319-EPYR_03343) organized in four operons encoding the T3SS machinery. The number and the arrangement of these genes are similar to the known *hrp *pathogenicity island found in *E. amylovora *[32], except for the absence of ORFU1 and ORFU2 which are located between the *hrpA *and *hrpS *genes in *E. amylovora*. Both ORFU1 and ORFU2 are also absent in the genome of *E. tasmaniensis *Et1/99 [16].

The genomic arrangement of the *hrp *region in *E. pyrifoliae *DSM 12163^T ^is identical to the T3SS arrangement found in *E. pyrifoliae *strain WT3 [33]. In *E. pyrifoliae *DSM 12163^T ^the *hrp *region is bordered by the *hrp *effector and elicitors region (HEE, EPYR_03311-EPYR_03318) and the Hrp-associated enzymes (HAE, EPYR_03344-EPYR_03349) region. Both regions are organized identically as in *E. amylovora *[12], while the HAE region is missing from the genome of the non-pathogenic *E. tasmaniensis *Et1/99 [16]. The HEE region includes the harpin genes *hrpN *and *hrpW*, *dspA/E *encoding a secreted effector essential for *E. amylovora *virulence [34], and the chaperone genes *orfA*, *orfB*, *orfC*, and *dspB/F*. The HAE region includes *hrp*-associated systemic virulence genes (*hsvABC*), the gene *hrpK *encoding for a putative type III translocator [35], and two genes encoding proteins of currently unknown function.

**Figure 4:**
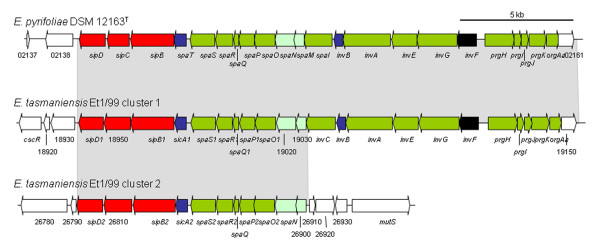
**Comparison of *inv*/*spa*-type Type III Secretion Systems in *E. pyrifoliae *DSM 12163^T ^and *E. tasmaniensis *Et1/99**. For the comparison, the annotation of the *E. tasmaniensis *Et1/99 clusters was manually checked. Previously denoted pseudogenes were shown to have close orthologs in *E. pyrifoliae *DSM 12163^T^, while "missing" genes in *E. tasmaniensis *Et1/99 [16] could be found. Blocks of related genes are shaded grey. Putative T3SS core genes are colored green, with low homology genes in light green. Effectors are colored red, regulatory genes black and chaperones blue. Genes with no homology are in white.

Genes of an *inv*/*spa *system are organized in a single gene cluster (EPYR_02139-EPYR_02160) in the genome of *E. pyrifoliae *DSM 12163^T ^(Fig. 4). This cluster displays similarity with those present in *E. amylovora *Ea273 [36], *E. tasmaniensis *Et1/99 [16], and the insect endosymbiont *Sodalis glossidinius *str. *morsitans *[28]. The highest sequence and structural similarity is to the PAI-3 system of *E. amylovora *Ea273 [36], where all the genes are also condensed into a single cluster. In *E. tasmaniensis *Et1/99, the *inv*/*spa *system is composed of two separate clusters complementing themselves to form a complete injection apparatus similar to that encoded by the *Salmonella typhimurium *pathogenicity island 1 [16]. Compared to *E. amylovora *Ea273 [36], only the hypothetical *orf43 *was absent in *E. pyrifoliae *DSM 12163^T^. The *inv/spa *system of *E. pyrifoliae *DSM 12163^T ^includes genes with no currently known function in plant pathogenicity or plant associations, and which are more related to T3SSs from endosymbionts and animal pathogens. It is proposed that *E. pyrifoliae *DSM 12163^T ^may utilize this second T3SS to facilitate vectoring associations with insects.

### Type VI secretion system

T6SS gene clusters have recently been found to be widespread among pathogenic and non-pathogenic Gram-negative bacteria [37]. Bacteria living in close association with eukaryotic cells are purported to use the T6SS as a mechanism for maintaining pathogenic or symbiotic interactions with their hosts. T6SS gene clusters consist of a set of 14 currently recognized core genes and conserved genes that vary in composition between species [38]. Two putative effector proteins in the human pathogen *Vibrio cholerae*, Hcp (haemolysin co-regulated protein) and Vgr (Val-Gly repeats), are secreted by a T6SS [39]. In the plant pathogenic bacterium *Pectobacterium atrosepticum *SCRI1043 (syn. *Erwinia carotovora *subsp. *atroseptica*), *vgr *and *hcp *genes are up-regulated when this pathogen is grown in the presence of potato extract, suggesting a potential role in virulence [40]. Two mutants, each defective in a gene of the T6SS gene cluster, showed reduced virulence compared to wild-type *P. atrosepticum *in potato tuber and stem virulence bioassays [41]. In the pea symbiont *Rhizobium leguminosarum*, a locus linked to the T6SS was discovered that negatively affects pea nodulation [42].

**Figure 5:**
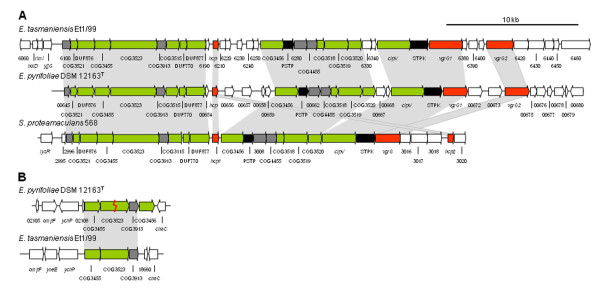
**Gene organization of Type VI Secretion System (T6SS) gene clusters**. (A) Large T6SS gene clusters. (B) Small T6SS gene clusters. Blocks of related genes are shaded grey. Putative core genes are colored green, putative effectors red, putative signal transducers black, conserved genes between clusters grey and genes without related or homologues in all other clusters white. The frameshift in EPYR_02110/EPYR_02111 is indicated by a red flash.

The T6SS gene cluster of *E. pyrifoliae *DSM 12163^T ^is composed of 30 genes (EPYR_00645-EPYR_00674; Fig. 5A), of which 15 were identified as core genes, four identified as conserved genes between species, two identified as putative signal transducers, and nine remaining as hypothetical genes. Conserved blocks of genes were observed among the different bacterial species, and were interspaced by hypothetical genes. Both *E. pyrifoliae *DSM 12163^T ^and *E. tasmaniensis *Et1/99 have closely related gene organizations with two exceptions: the genes between *hcp *and COG3456 are different (EPYR_00656-EPYR_00659, ETA_06210-06250 respectively) and the VgrG2 of *E. pyrifoliae *DSM 12163^T ^is more closely related to VgrG1 of *E. tasmaniensis *Et1/99. These genes (EPYR_00656-EPYR_00659) have a lower G+C content than the backbone of the chromosome, indicating an acquired foreign origin of these genes. The genes downstream of *vgrG2 *in *E. pyrifoliae *DSM 12163^T ^are homologous to genes downstream of *vgrG1 *in *E. tasmaniensis *Et1/99 (EPYR_00676, EPYR_00677, EPYR_00679, ETA_06380-06400).

The Hcp encoded in the T6SS cluster of *E. pyrifoliae *DSM 12163^T ^is 91.9% identical to its ortholog in *E. tasmaniensis *Et1/99. Apart from this *hcp *gene, *E. pyrifoliae *DSM 12163^T ^possesses two additional *hcp *genes on the plasmids pEP3 and pEP5 (Fig. 2). The Hcp proteins encoded on the *E. pyrifoliae *DSM 12163^T ^plasmids are more related to each other (82.5% identity) than to the Hcp proteins of the large chromosomal T6SS clusters. The related enterobacterium *Serratia proteamaculans *568 has a similar gene organization for its T6SS (Fig. 5A), but has no additional genes downstream of *hcp1*, an additional *hcp *gene (*hcp2*), and only one *vgrG *gene. In comparison, Hcp1 is more closely related to the Hcp genes of *E. pyrifoliae *DSM 12163^T ^and *E. tasmaniensis *Et1/99 than to Hcp2. The *P. atrosepticum *SCRI1043 T6SS gene cluster is completely different from that in *E. pyrifoliae *DSM 12163^T ^regarding its organization [37,43].

A second set of designated T6SS-related genes was found in the genomes of *E. pyrifoliae *DSM 12163^T ^(EPYR_02109-EPYR_02113) and *E. tasmaniensis *Et1/99 (ETA_18657-ETA_18659) (Fig. 5B) which are absent in *S*. *proteamaculans *568. In *E. pyrifoliae *DSM 12163^T^, three putative core genes (EPYR_02109, EPYR_02110/02111, EPYR_02113) and one conserved gene were found (EPYR_02112), while in *E. tasmaniensis *Et1/99 one of these core genes was absent. Genes in this second set show low homology to each other and to the large T6SS clusters. A frameshift in COG3523 (EPYR_02110/02111) within this T6SS cluster of *E. pyrifoliae *DSM 12163^T ^was identified which leads to an early stop-codon in the gene. It can be deduced that this gene cluster is inactivated, and prone to mutations and deletions.

### Flagellar genes

Two sets of genes encoding for flagellar assembly and chemotaxis related proteins were found in the genome of *E. pyrifoliae *DSM 12163^T^. One set is tightly clustered (EPYR_00976-EPYR_01028) and the encoded proteins show higher identity with the corresponding proteins of *Salmonella *and *Escherichia *spp. than with those of *E. tasmaniensis *Et1/99 [16]. This suggests that the entire region was acquired as a genomic island via horizontal genetic transfer. Only the right boundary of the genomic island is found in *E. pyrifoliae *DSM 12163^T^, as discernible by a reduced G+C-content bordered by an IS2-type integrase (EPYR_01040) flanking *smpB *(EPYR_01041), a gene also found the *E. tasmaniensis *Et1/99 genome (ETA_09720). The left border of the genomic island does not display distinctive insertion features. The first upstream-gene with high homology to *E. tasmaniensis *Et1/99 is *tsx *(EPYR_00974), which matches a gene that encodes a nucleoside-specific channel-forming protein in *E. tasmaniensis *Et1/99 (ETA_09440). Notably, in *E. tasmaniensis *Et1/99 the region included between ETA_09440 and ETA_09720 contains quorum-sensing genes *expRI *which are absent in *E. pyrifoliae *DSM 12163^T^.

A second complete set of flagellar genes is present but split among different clusters in the genome of *E. pyrifoliae *DSM 12163^T ^(EPYR_01609-EPYR_01614, EPYR_01651-EPYR_01668, EPYR_02267-EPYR_02282, EPYR_02322-EPYR_02336). This second set closely resembles the flagellar genes found in *E. tasmaniensis *Et1/99 and appears to be ancestral in *E. pyrifoliae *DSM 12163^T^. Compared to the acquired flagellar assembly apparatus, the native gene cluster contains two extra genes (EPYR_01615 and EPYR_02267) encoding FliZ, a putative alternative sigma factor regulatory protein and the putative CheV chemotaxis signal transduction protein, respectively.

### Sorbitol metabolism

Metabolism of sorbitol has been described as a contributing virulence factor in *E. amylovora *[44] on pome fruits which utilize sorbitol as a primary carbon storage compound [45]. The genome of *E. pyrifoliae *DSM 12163^T ^contains a complete sorbitol gene cluster (EPYR_00602-EPYR_00606), and the organism is able to utilize sorbitol as a sole carbon source [19]. The non-pathogenic *E. tasmaniensis *Et1/99 lacks a sorbitol gene cluster in its genome [16], and may thus be disadvantaged in competitive interactions in environments rich in sorbitol. The exact role of sorbitol utilization in virulence is unresolved, since sorbitol content in apple trees has no specific effect on fire blight disease severity [46]. Associations between sorbitol and fire blight [47] suggest rather an osmotic-potential effect which could be due to various carbon compounds. Transgenic apple trees with down-regulated sorbitol synthesis compensate by increasing production of other sugars (i.e., sucrose, glucose) [45], and both of these are also substrates for *E. pyrifoliae *and *E. amylovora *which carry the respective metabolic genes.

### Exopolysaccharide biosynthesis

**Figure 6:**
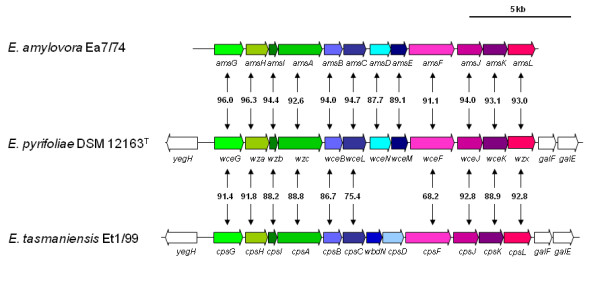
**Comparison of the exopolysaccharide (EPS) biosynthetic clusters of *E. pyrifoliae *DSM 12163^T ^(middle), *E. amylovora *Ea7/74 (top) and *E. tasmaniensis *Et1/99 (bottom)**. Identical colours indicate identical predicted functions. White arrows indicate flanking genes probably not involved in EPS biosynthesis. The numbers between the gene clusters indicate the sequence identity of the translated gene products indicated.

*E. pyrifoliae *produces an exopolysaccharide (EPS) that is similar to amylovoran produced by *E. amylovora *[48]. In *E. amylovora*, mutation of one of the exopolysaccharide genes, *wceB*, impaired pathogen EPS biosynthesis and reduced virulence on apple [48]. The EPS biosynthetic gene cluster of *E. pyrifoliae *DSM 12163^T ^(EPYR_01479-EPYR_01490) is more related to the gene cluster of *E. amylovora *Ea7/74 [49] than that of *E. tasmaniensis *Et1/99 [16]. The latter has different glycosyltransferases in the central part of the cluster (WceNM; Fig. 6) and is more similar to the stewartan biosynthetic cluster found in *P. stewartii *subsp. *stewarii *DC283 [50]. Overall, proteins encoded by the EPS cluster found in *E. pyrifoliae *DSM 12163^ T ^are 87.7 - 96.3% identical to the corresponding proteins in *E. amylovora *Ea7/74, while the identities to *E. tasmaniensis *Et1/99 range between 68.2 and 92.8%, with no match for WceN and WceM (Fig. 6). Thus the *E. pyrifoliae *amylovoran genes are anticipated to have a role in virulence similar to that previously demonstrated in *E. amylovora*.

A second EPS produced by *E. amylovora *strains, levan, is also reported to contribute to virulence [51]. *E. pyrifoliae *DSM 12163^T ^does not have levan biosynthetic genes and does not produce levan, which has also been reported to be lacking in the related fire blight *Erwinia *sp. from Japan [9]. It is proposed that the lack of levan may contribute to the restricted host-range in these species compared to *E. amylovora*, although levan biosynthesis *in vitro *has been reported in non-pathogenic *E. tasmaniensis *Et1/99.

### Quorum-sensing apparatus

Quorum-sensing (QS) refers to the ability of bacteria to regulate gene expression in accordance with the presence of extracellular signalling molecules, or autoinducers (AI), that are produced in a cell density-dependent manner [52]. Two principal QS systems are known in Gram-negative bacteria and are defined by the chemical nature of the AI involved [53].

The AI-1 system utilizes *N*-acyl homoserine lactones (AHLs), produced by the LuxI family of proteins, as signal molecules. This system was first described in the marine bacterium *Vibrio fisheri *[54], where it controls bioluminescence production. Above a certain concentration, the secreted AHL binds the LuxR receptor and the resulting complex activates target gene transcription by binding a specific promoter site (*lux *box). Several AHLs have since been described in a wide-variety of Gram-negative pathogenic and non-pathogenic bacteria, differing principally in the *N*-acyl side chain moiety of the AHLs, with each unique AHL signal controlled by its own pair of *luxRI *gene orthologs [55].

In *E. pyrifoliae*, proteins encoded by one gene set (EPYR_02385-EPYR_02386) have low sequence identity with the autoinducer biosynthetic and receptor proteins PhzI/PhzR of *Pseudomonas chlororaphis *(22 and 26% respectively) and ExpI/ExpR of *P. atrosepticum *SCRI 1043 (17% and 22%). However, EPYR_02386 also shows 45% identity with the DNA-binding transcriptional activator SdiA, which regulates cell division in *Escherichia coli *and *S. typhimurium *[56], and which is known to respond to AHLs generated by other microbial species. For SdiA, no cognate gene encoding a corresponding signal-generating enzyme is present in either *E. coli *or in *S. typhimurium *[57]. Thus, SdiA may have a similar activity/non-activity in *E. pyrifoliae *and other *Erwinia *species.

Given the very low identity of EPYR_02385 to known AHL synthesis protein and the failure of *E. pyrifoliae *DSM 12163^T ^to induce a positive reaction in AHL-biosensors *Chromobacterium violaceum *CV026 and *Agrobacterium tumefaciens *NTL/pZLR4 (data not shown), it is hypothesized that the gene set composed of EPYR_02385 and EPYR_02386 contributes to production and detection of a yet unknown extracellular signal not obviously related to known QS signals.

A second QS system, based on production of an AI-2 signal molecule and controlled by the LuxS protein, is also widespread among Gram-negative and Gram-positive bacteria [58], and putatively involved in cross-species bacterial communication [59]. LuxS, a S-ribosylhomocysteine lyase, is however also known to be a central component of the activated methyl cycle (AMC), a metabolic cycle responsible for recycling methionine and generation of the major methyl donor S-adenosyl-L-methionine (SAM) in bacterial cells [60,61]. It is thus critical that the decision of whether or not the AI-2 system is present in certain species is not only based on the presence of *luxS*, but also on presence and functionality of the genes coding for the AI-2 receptors [61]. *E. pyrifoliae *DSM 12163^T ^has a functional *luxS *gene (EPYR_03027) but it lacks orthologs for both of the two known AI-2 receptors: the LuxPQ-receptor of *Vibrio harveyi *[62] and the Lsr ABC-transporter of *Salmonella typhimurium *[63]. Thus, as in *E. amylovora *Ea273 [64], which also carries *luxS *but lacks AI-2 receptors, this gene probably has a metabolic rather than QS role in *E. pyrifoliae*.

### Iron uptake determinants

Iron is an essential nutritional factor for plant pathogenic bacteria, which have high-affinity iron acquisition systems in order to supply this need [65]. Iron regulation of a wide range of genes is coordinated by Fur (encoded by EPYR_02663), which specifically regulates biosynthesis and uptake of iron-affinity siderophores [66]. The genome of *E. pyrifoliae *DSM 12163^T ^contains four TonB-dependent receptor genes, one of which is also a putative copper receptor (*oprC*; EPYR_01920).

Another TonB-dependent receptor gene (EPYR_03492) encodes an ortholog of the *E. amylovora *ferrioxamine-receptor FoxR [67]. The gene product is directly involved in uptake of ferrioxamines into the periplasm. In *E. amylovora*, *foxR *mutants retain ability to synthesize desferrioxamine E, but lose their ability to utilize this as an iron substrate, are impaired in growth under iron-limited conditions such as found on flower surfaces, have reduced virulence, and are less resistant to plant defence responses [67,68]. Adjacent to *foxR*, *E. pyrifoliae *also has a biosynthetic gene cluster *dfoJAC *(EPYR_03489-EPYR_03491) containing an ortholog of *dfoA*, one of the biosynthetic genes for desferrioxamine in *E. amylovora *CFBP1430 [67], indicating that *E. pyrifoliae *DSM 12163^T ^synthesizes desferrioxamine E and has an iron-acquisition system similar to *E. amylovora *[69]. In *E. pyrifoliae *DSM 12163^T^, a ferrihydroxamate TonB-dependent receptor and ABC transport system *fhuACDB*; EPYR_00903-EPYR_00906) is also present, which may contribute to uptake of ferrihydroxamate siderophores (Stockwell et al. 2008).

A fourth TonB-dependent receptor (EPYR_01969) found in *E. pyrifoliae *DSM 12163^T ^is predicted to belong to the Transport Classification DataBase (TCDB; http://www.tcdb.org/) group TC 1.B.14.2.-, which consists primarily of heme and porphyrin uptake systems. An inactivated homolog of the TonB-dependent receptor *yncD *(EPYR_02731-EPYR_02732) is also present in the *E. pyrifoliae *DSM 12163^T ^genome, but is inactivated by frameshifts. The *ent-fep-fec *gene cluster encoding enterobactin biosynthesis and transport in many *Enterobacteriaceae*, is absent in the genome of *E. pyrifoliae *DSM 12163^T^, as has been observed for *E. tasmaniensis *Et1/99 [16].

### Comparative genomics to non-pathogenic *Erwinia tasmaniensis *Et1/99

**Figure 7:**
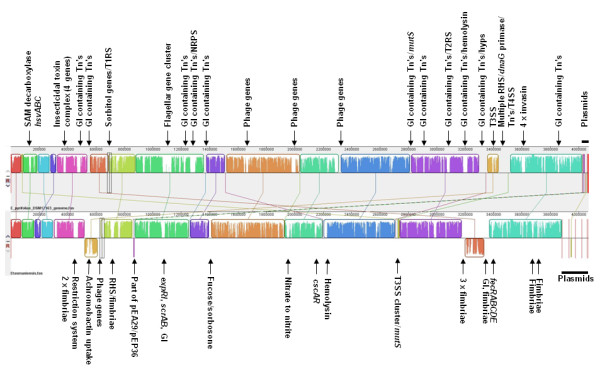
**Mauve progressive alignment of the genomes of *E. pyrifoliae *DSM 12163^T ^(top) and *E. tasmaniensis *Et1/99 (bottom)**. Some relevant features within regions that have large differences in the alignment are indicated with arrows, plasmids are indicated as horizontal bars. Abbreviations: GI: genomic islands; T1RS: type 1 restriction system; T2RS: type 2 restriction sytem; Tn: transposase, T3SS: type 3 secretion system, T4SS: type 4 secretion system.

The chromosome of *E. tasmaniensis *Et1/99 is 3,883,467 bp [16], compared to the 4,026,286 bp chromosome of *E. pyrifoliae *DSM 12163^T^. Genomic comparison of the complete genomes of the two related bacteria was performed using a Mauve progressive alignment [70], and large gaps were examined against the annotation (Fig. 7). With the exception of the plasmids, the two genomes are essentially collinear, with only a few genomic blocks exchanged.

The difference in genome size between *E. pyrifoliae *DSM 12163^T ^and *E. tasmaniensis *Et1/99 is largely due to the insertion of mobile genetic elements in *E. pyrifoliae *DSM 12163^T^. At least three regions contain phage genes, indicating insertion of prophages or remnants thereof. Additionally, a large number of genomic islands have inserted into the genome of *E. pyrifoliae *DSM 12163^T^, as indicated by the large number of possibly active or inactivated transposases identified. Of the remaining areas, two regions encode restriction systems and one cluster carries a complete additional set of flagellar genes (EPYR_00976-EPYR_01028; see above).

In contrast, *E. tasmaniensis *Et1/99 contains a large number of fimbrial gene clusters spread across the chromosome that are lacking in *E. pyrifoliae *DSM 12163^T^. Additionally, the *nas/nir *cluster is present in *E. tasmaniensis *but absent in *E. pyrifoliae *DSM 12163^T^, which explains the inability of *E. pyrifoliae *to generate nitrite from nitrate [7,19]. We observed that the chromosome of *E. tasmaniensis *Et1/99 encodes part of the central region of *E. pyrifoliae *plasmid pEP36 carrying the *thiOSGF *and the *betB *genes, but not the entire plasmid. *E. tasmaniensis *Et1/99 also carries genes for the ferric citrate uptake system *fecRABCDE *and for achromobactin uptake, which are all lacking in *E. pyrifoliae *DSM 12163^T^. From the alignment (Fig. 7), it was observed that *E. pyrifoliae *DSM 12163^T ^contains the same set of T6SS genes as found in *E. tasmaniensis *Et1/99 but not previously reported. The incomplete *inv*/*spa *T3SS cluster, located close to the *mutS *gene in *E. tasmaniensis *Et1/99 (Fig. 4), is absent in *E. pyrifoliae *DSM 12163^T^.

Overall, the large-scale analysis of genomic differences between *E. pyrifoliae *DSM 12163^T ^and *E. tasmaniensis *Et1/99 does not clearly reveal why *E. pyrifoliae *and not *E. tasmaniensis *is pathogenic. Mutational analysis of different genomic features is needed to determine their role in pathogenicity compared with non-pathogenic *E. tasmaniensis*. Our results also suggest that comparison of the genome sequences of *E. pyrifoliae *and *E. amylovora*, and different strains within each species, could reveal host-range determinants.

## Conclusions

Compared to *E. amylovora*, the genome of *E. pyrifoliae *DSM 12163^T ^encodes many of the same virulence factors, including two T3SSs, sorbitol metabolism, exopolysaccharides, and desferrioxamine biosynthesis. However, in *E. pyrifoliae *levan production and a third T3SS cluster are absent. Whether these factors contribute to the reduced host-range of this pathogen remains to be elucidated. Comparison to the genome of the non-pathogenic *E. tasmaniensis *Et1/99 indicated an additional flagellar gene cluster, absence of AHL biosynthesis genes, and a modified range of iron acquisition systems which may play a role in pathogenicity. As more of the species that have been identified with fire blight-like symptoms or as epiphytes on *Rosaceae *[9,13,18,36,71-77] are sequenced and can be compared on a whole-genome scale, further clues to the evolution and origin of necrotrophic *Erwinia*, and insights into host-pathogen interactions, can be anticipated.

## Methods

### Whole-genome sequencing

Genomic DNA was isolated using the Wizard Genomic DNA Purification Kit (Promega, Madison WI, USA). Whole-genome sequencing was performed by GATC (Konstanz, Germany) using both a 454 GS-FLX and a Solexa sequencer.

From a single run with the 454 GS-FLX, a total of 533,966 high-quality filtered sequence reads were generated with an average read length of 232 bp (Table 2). Coverage was equivalent to 31 times. Quality filtered sequences from whole-genome shotgun-sequencing were assembled using the 454 Newbler assembler. In total, 727 contigs were generated, of which 171 contigs larger than 500 bp. The average size of the large contigs was 5,480 bp. Reassembly from the individual data sets of each of the regions on the PicoTitrePlate (Roche) generated less contigs that were longer at average, and assembly of all contig sets allowed the exclusion of bad or contaminant sequences, that mainly constituted the majority of small contigs, under the assumption that the newly generated contigs should be covered by assemblies from all data sets.

**Table 2:** Overview of sequence data for the genome of *E. pyrifoliae *DSM 12163^T^.

Sequencing method	454 sequencing	Solexa
Total reads	533,966	4,835,468
Total sequence output	124,139,960 bp	174,076,848 bp
Average read length	232 bp	36 bp
Coverage (4 Mb)	31×	43.5×
No. of contigs^a^	727	
No. of contigs > 500 bp	171	
Total size	3,874,928	
Average size	22.7 kb	

^a ^assembly only possible with 454 reads.

A total of 4,835,468 high-quality filtered sequence reads with an average read length of 36 bp were generated by Solexa sequencing (Table 2). Average coverage was equivalent to 43.5×. Reads were assembled using the *E. pyrifoliae *Ep1/96 chromosome, plasmid pEP36 and pEP2.6. All reads were additionally *de novo *assembled using the program EDENA [78] with manually optimized settings. This yielded 3,162 contigs (N_50 _= 22,267; mean length = 1,206; maximum length = 12,612; minimum length = 100). The assembled contigs were aligned to the sequence using the SeqMan program of the LASERGENE package (DNASTAR, Madison, WI, USA).

### Assembly

Assembly of the *E. pyrifoliae *DSM12163^T ^genome sequence was done in two steps. For the chromosome, GenBank accession FP236842 was used as reference. The sequences for plasmids pEP36 and pEP2.6 (AY123045, AY123048) were published [8], and used as a template for assembling these sequences. In a first step, the longer pyrosequencing reads were mapped to the available references using Roche's gsMapper application. When both sequence coverage and quality indicated a difference between the two strains with high confidence, the reference sequence was modified to reflect this change. Afterwards, both 454 and the shorter Solexa reads were mapped to the modified sequences with several custom scripts. In most cases, the Solexa reads supported the information from the 454 sequences and in some cases the Solexa data could be used to close gaps in the references where no 454 data was available. Based on the mapping information from both 454 and Solexa reads, contigs were created where coverage was high enough and at least 75% of all mapped reads agreed about a base for a given position. When no consensus base could be determined, an 'N' was inserted.

The reference sequence was also used as a template for the previously assembled contigs using SeqMan. Most contigs aligned to the template, and the non-aligned contigs were mainly due to false assembly in an earlier phase and could be resolved by realignment. The leftover contigs were checked by BlastN versus the template, and two contigs that were not covered by any part of the template were extended by several rounds of assembly versus the 454 reads until they were closed. This assembly was then confirmed by assembly using the Solexa reads, and by shifting the zero-point in the newly assembled plasmid sequences (pEP5 and pPE3).

### Genome annotation

Genes were predicted using a combined strategy [79] based on the CDS prediction programs Glimmer [80] and Critica [81]. Subsequently, the potential function of each predicted gene was automatically assigned using the GenDB annotation pipeline [17]. The resulting genome annotation was curated manually, and metabolic pathways were identified using the KEGG pathways [82] tool in GenDB.

### Software

Routine sequence manipulations were done using several subroutines of the LASERGENE package. Whole-genome comparisons were done using the progressive alignment option of the Mauve comparison software (Version 2.0 [70]).

For the detailed analysis of the *E. pyrifoliae *DSM 12163^T ^genome sequence, the Transport Classification DataBase (TCDB) http://www.tcdb.org/ was used for improving the automated annotation of transporters. For comparative classification of the T3SS effectors, the *Pseudomonas syringae *Hop database http://pseudomonas-syringae.org/Hop_database.xls was blasted on protein level versus the annotation of the *E. pyrifoliae *DSM 12163^ T ^genome.

### Accession numbers

The genome sequence of *E. pyrifoliae *DSM 12163^T ^has been deposited at EBI and received the accession numbers EMBL:FN392235 (chromosome), EMBL:FN392238 (pEP36), EMBL:FN392239 (pEP5), EMBL:FN392237 (pEP3) and EMBL:FN392236 (pEP2.6).

## Authors' contributions

THMS and SJ conducted the genome assembly. THMS performed the annotation, comparative genomics and wrote the manuscript. SJ, AG, FR and TK participated in the genome annotation, pathway identification and in writing the manuscript. JEF contributed to the project conception. BD conceived of and supervised the project and participated in writing the manuscript. All authors read and approved the final manuscript.

## References

[B1] Duffy B Schärer H-J Bünter M Klay A Holliger E Regulatory measures against *Erwinia amylovora *in Switzerland EPPO Bull 2005 35 239 244 10.1111/j.1365-2338.2005.00820.x

[B2] Bonn WG Zwet T van der Vanneste JL Distribution and economic importance of fire blight Fire blight the disease and its causative agent, Erwinia amylovora 2000 Wallingford, UK CAB International 37 53 full_text

[B3] Jock S Donat V López MM Bazzi C Geider K Following spread of fire blight in Western, Central and Southern Europe by molecular differentiation of *Erwinia amylovora *strains with PFGE analysis Environ Microbiol 2002 4 2 106 114 10.1046/j.1462-2920.2002.00277.x 11972620

[B4] Thomson SV Vanneste JL Epidemiology of fire blight Fire blight the disease and its causative agent, Erwinia amylovora 2000 Wallingford, UK CAB International 9 36 full_text

[B5] Johnson KB Stockwell VO Management of fire blight a case study in microbial ecology Annu Rev Phytopathol 1998 36 227 248 10.1146/annurev.phyto.36.1.227 15012499

[B6] Geider K Auling G Jakovljevic V Völksch B A polyphasic approach assigns the pathogenic *Erwinia *strains from diseased pear trees in Japan to *Erwinia pyrifoliae* Lett Appl Microbiol 2009 48 324 330 10.1111/j.1472-765X.2008.02535.x 19187512

[B7] Kim W-S Gardan L Rhim S-L Geider K *Erwinia pyrifoliae *sp. nov., a novel pathogen that affects Asian pear trees (*Pyrus pyrifolia *Nakai) Int J Syst Bacteriol 1999 49 899 906 10319516 10.1099/00207713-49-2-899

[B8] McGhee GC Schnabel EL Maxson-Stein K Jones B Stromberg VK Lacy GH Jones AL Relatedness of chromosomal and plasmid DNAs of *Erwinia pyrifoliae *and *Erwinia amylovora* Appl Environ Microbiol 2002 68 12 6182 6192 10.1128/AEM.68.12.6182-6192.2002 12450843 PMC134437

[B9] Kim W-S Hildebrand M Jock S Geider K Molecular comparison of pathogenic bacteria from pear trees in Japan and the fire blight pathogen *Erwinia amylovora* Microbiology 2001 147 2951 2959 11700346 10.1099/00221287-147-11-2951

[B10] Lehman SM Kim W-S Castle AJ Svircev AM Duplex real-time polymerase chain reaction reveals competition between *Erwinia amylovora *and *E. pyrifoliae *on pear blossoms Phytopathology 2008 98 6 673 679 10.1094/PHYTO-98-6-0673 18944291

[B11] Eastgate JA *Erwinia amylovora *the molecular basis of fireblight disease Mol Plant Pathol 2000 1 6 325 329 10.1046/j.1364-3703.2000.00044.x 20572979

[B12] Oh C-S Beer SV Molecular genetics of *Erwinia amylovora *involved in the development of fire blight FEMS Microbiol Lett 2005 253 185 192 10.1016/j.femsle.2005.09.051 16253442

[B13] Triplett LR Zhao Y Sundin GW Genetic differences between blight-causing *Erwinia *species with differing host specificities, identified by suppression subtractive hybridization Appl Environ Microbiol 2006 72 11 7359 7364 10.1128/AEM.01159-06 16963554 PMC1636173

[B14] Shrestha R Lee SH Kim JE Wilson C Choi S-G Park DH Wang MH Hur JH Lim CK Diversity and detection of Korean *Erwinia pyrifoliae *strains as determined by plasmid profiling, phylogenetic analysis and PCR Plant Pathol 2007 56 1023 1031 10.1111/j.1365-3059.2007.01679.x

[B15] Shrestha R Tsuchiya K Baek SJ Bae HN Hwang I Hur JH Lim CK Identification of *dspEF, hrpW *and *hrpN *loci and characterization of the *hrpN*_Ep _gene in *Erwinia pyrifoliae* J Gen Plant Pathol 2005 71 211 220 10.1007/s10327-005-0191-6

[B16] Kube M Migdoll AM Müller I Kuhl H Beck A Reinhardt R Geider K The genome of *Erwinia tasmaniensis *strain Et1/99, a non-pathogenic bacterium in the genus *Erwinia* Environ Microbiol 2008 10 9 2211 2222 10.1111/j.1462-2920.2008.01639.x 18462403

[B17] Meyer F Goesmann A McHardy AC Bartels D Bekel T Clausen J Kalinowski J Linke B Rupp O Giegerich R GenDB - an open source genome annotation system for prokaryote genomes Nucleic Acids Res 2003 31 2187 2195 10.1093/nar/gkg312 12682369 PMC153740

[B18] Maxson-Stein K McGhee GC Smith JJ Jones AL Sundin GW Genetic analysis of a pathogenic *Erwinia *sp. isolated from pear in Japan Phytopathology 2003 93 11 1393 1399 10.1094/PHYTO.2003.93.11.1393 18944067

[B19] Rhim S-L Völksch B Gardan L Paulin J-P Langlotz C Kim W-S Geider K *Erwinia pyrifoliae *an Erwinia species different from *Erwinia amylovora *causes a necrotic disease of Asian pear trees Plant Pathol 1999 48 514 520 10.1046/j.1365-3059.1999.00376.x

[B20] Fu J-F Chang H-C Chen Y-M Chang Y-S Liu S-T Sequence analysis of an *Erwinia stewartii *plasmid, pSW100 Plasmid 1995 34 2 75 84 10.1006/plas.1995.9999 8559805

[B21] Lonetto M Gribskov M Gross CA The s^70 ^family sequence conservation and evolutionary relationships J Bacteriol 1992 174 12 3843 3849 1597408 10.1128/jb.174.12.3843-3849.1992 PMC206090

[B22] Anderson M Pollitt CE Roberts IS Eastgate JA Identification and characterization of the *Erwinia amylovora rpoS *gene RpoS is not involved in induction of fireblight disease symptoms J Bacteriol 1998 180 6789 6792 9852034 10.1128/jb.180.24.6789-6792.1998 PMC107793

[B23] Andersson RA Kõiv V Norman-Setterblad C Pirhonen M Role of RpoS in virulence and stress tolerance of the plant pathogen *Erwinia carotovora *subsp. *carotovora* Microbiology 1999 145 3547 3556 10627052 10.1099/00221287-145-12-3547

[B24] Mukherjee A Cui Y Ma W Liu Y Ishihama A Eisenstark A Chatterjee AK RpoS (Sigma-S) controls expression of *rsmA*a global regulator of secondary metabolites, harpin and extracellular proteins in *Erwinia carotovora* J Bacteriol 1998 180 3629 3634 9658007 10.1128/jb.180.14.3629-3634.1998 PMC107332

[B25] Hueck CJ Type III protein secretion systems in bacterial pathogens of animals and plants Microbiol Mol Biol Rev 1998 62 2 379 433 9618447 10.1128/mmbr.62.2.379-433.1998 PMC98920

[B26] Freiberg C Fellay R Bairoch A Broughton WJ Rosenthal A Perret X Molecular basis of symbiosis between *Rhizobium *and legumes Nature 1997 387 394 401 10.1038/387394a0 9163424

[B27] Göttfert M Röthlisberger S Kündig C Beck C Marty R Hennecke H Potential symbiosis-specific genes uncovered by sequencing a 410-kilobase DNA region of the *Bradyrhizobium japonicum *chromosome J Bacteriol 2001 183 4 1405 1412 10.1128/JB.183.4.1405-1412.2001 11157954 PMC95015

[B28] Dale C Young SA Haydon DT Welburn SC The insect endosymbiont *Sodalis glossinidius *utilizes a type III secretion system for cell invasion Proc Natl Acad Sci USA 2001 98 4 1883 1888 10.1073/pnas.021450998 11172045 PMC29351

[B29] Alfano JR Collmer A Type III secretion system effector proteins double agents in bacterial disease and plant defence Annu Rev Phytopathol 2004 42 385 414 10.1146/annurev.phyto.42.040103.110731 15283671

[B30] Jock S Kim W-S Barny M-A Geider K Molecular characterization of natural *Erwinia pyrifoliae *strains deficient in hypersensitive response Appl Environ Microbiol 2003 69 1 679 682 10.1128/AEM.69.1.679-682.2003 12514060 PMC152409

[B31] McClelland M Sanderson KE Spieth J Clifton SW Latreille P Courtney L Porwollik S Ali J Dante M Du F Complete genome sequence of *Salmonella enterica *serovar Typhimurium LT2 Nature 2001 413 852 856 10.1038/35101614 11677609

[B32] Oh C-S Kim JF Beer SV The Hrp pathogenicity island of *Erwinia amylovora *and identification of three novel genes required for systemic infection Mol Plant Pathol 2005 6 2 125 138 10.1111/j.1364-3703.2005.00269.x 20565644

[B33] Shrestha R Lee SH Cho S Lim CK Hong S Cho JM Hwang I Hur JH Kim W-S The *hrp *genes cluster in *Erwinia pyrifoliae *and determination of HR active domain in HrpN_Ep _protein Acta Hort 2008 793 221 230 18319611

[B34] Boureau T ElMaarouf-Bouteau H Garnier A Brisset M-N Perino C Pucheu I Barny M-A DspA/E, a type III effector essential for *Erwinia amylovora *pathogenicity and growth in planta, induces cell death in host apple and nonhost tobacco plants Mol Plant-Microbe Interact 2006 19 1 16 24 10.1094/MPMI-19-0016 16404949

[B35] Petnicki-Ocwieja T van Dijk K Alfano JR The *hrpK *operon of *Pseudomonas syringae *pv. tomato DC3000 encodes two proteins secreted by the type III (Hrp) protein secretion system HopB1 and HrpK, a putative type III translocator J Bacteriol 2005 187 2 649 663 10.1128/JB.187.2.649-663.2005 15629936 PMC543549

[B36] Bocsanczy AM Beer SV Perna NT Biehl B Glasner JD Cartinhour SW Schneider DJ DeClerck GA Sebaihia M Parkhill J Contributions of the genome sequence of *Erwinia amylovora *to the fire blight community Acta Hort 2008 793 163 170

[B37] Boyer F Fichant G Berthod J Vandenbrouck Y Attree I Dissecting the bacterial type VI secretion system by a genome wide in silico analysis what can be learnt from available microbial genomic resources? BMC Genomics 2009 10 104 10.1186/1471-2164-10-104 19284603 PMC2660368

[B38] Bingle LEH Bailey CM Pallen MJ Type VI secretion a beginner's guide Curr Opin Microbiol 2008 11 1 3 8 10.1016/j.mib.2008.01.006 18289922

[B39] Pukatzki S Ma AT Sturtevant D Krastins B Sarracino D Nelson WC Heidelberg JF Mekalanos JJ Identification of a conserved bacterial protein secretion system in *Vibrio cholerae *using the *Dictyostelium *host model system Proc Natl Acad Sci USA 2006 103 5 1528 1533 10.1073/pnas.0510322103 16432199 PMC1345711

[B40] Mattinen L Nissinen R Riipi T Kalkkinen N Pirhonen M Host-extract induced changes in the secretome of the plant pathogenic bacterium *Pectobacterium atrosepticum* Proteomics 2007 7 3527 3537 10.1002/pmic.200600759 17726675

[B41] Liu H Coulthurst SJ Pritchard L Hedley PE Ravensdale M Humphris S Burr T Takle G Brurberg M-B Birch PRJ Quorum sensing coordinates brute force and stealth modes of infection in the plant pathogen *Pectobacterium atrosepticum* PLoS Pathog 2008 4 6 e1000093 10.1371/journal.ppat.1000093 18566662 PMC2413422

[B42] Roest HP Mulders IHM Spaink HP Wijffelman CA Lugtenberg BJJ A *Rhizobium leguminosarum *biovar *trifolii *locus not localized on the Sym plasmid hinders effective nodulation on plants of the pea cross-inoculation group Mol Plant-Microbe Interact 1997 10 7 938 941 10.1094/MPMI.1997.10.7.938 9304865

[B43] Bell KS Sebaihia M Pritchard L Holden MTG Hyman LJ Holeva MC Thomson NR Bentley SD Churcher LJC Mungall K Genome sequence of the enterobacterial phytopathogen *Erwinia carotovora *subsp *atroseptica *and characterization of virulence factors Proc Natl Acad Sci USA 2004 101 30 11105 11110 10.1073/pnas.0402424101 15263089 PMC503747

[B44] Aldridge P Metzger M Geider K Genetics of sorbitol metabolism in *Erwinia amylovora *and its influence on bacterial virulence Mol Gen Genet 1997 256 6 611 619 10.1007/s004380050609 9435786

[B45] Teo G Suzuki Y Uratsu SL Lampinen B Ormonde N Hu WK DeJong TM Dandekar AM Silencing leaf sorbitol synthesis alters long-distance partitioning and apple fruit quality Proc Natl Acad Sci USA 2006 103 49 18842 18847 10.1073/pnas.0605873103 17132742 PMC1693749

[B46] Duffy B Dandekar AM Sorbitol has no role in fire blight as demonstrated using transgenic apple with constitutively altered content Acta Hort 2008 793 279 283

[B47] Suleman P Steiner PW Relationship between sorbitol and solute potential in apple shoots relative to fire blight symptom development after infection by *Erwinia amylovora* Phytopathology 1994 84 1244 1250 10.1094/Phyto-84-1244

[B48] Kim W-S Schollmeyer M Nimtz M Wray V Geider K Genetics of biosynthesis and structure of the capsular exopolysaccharide from the Asian pear pathogen *Erwinia pyrifoliae* Microbiology 2002 148 4015 4024 12480905 10.1099/00221287-148-12-4015

[B49] Bernhard F Schullerus D Bellemann P Geider K Nimtz M Majerczak DR Coplin DL Genetics and complementation of DNA regions involved in amylovoran synthesis of *Erwinia amylovora *and stewartan synthesis of *Erwinia stewartii* Acta Hort 1996 411 269 274

[B50] Coplin DL Majerczak DR Bugert P Geider K Nucleotide sequence analysis of the *Erwinia stewartii cps *gene cluster for synthesis of stewartan and comparison to the *Erwinia amylovora ams *cluster for synthesis of amylovoran Acta Hort 1996 411 251 257

[B51] Gross M Geier G Rudolph K Geider K Levan and levansucrase synthesized by the fireblight pathogen *Erwinia amylovora* Physiol Mol Plant Pathol 1992 40 6 371 381 10.1016/0885-5765(92)90029-U

[B52] Pierson LS III Wood DW Pierson EA Homoserine lactone-mediated gene regulation in plant-associated bacteria Annu Rev Phytopathol 1998 36 207 225 10.1146/annurev.phyto.36.1.207 15012498

[B53] Miller MB Bassler BL Quorum sensing in bacteria Annu Rev Microbiol 2001 55 165 199 10.1146/annurev.micro.55.1.165 11544353

[B54] Nealson KH Hastings JW Bacterial bioluminescence its control and ecological significance Microbiol Rev 1979 43 4 496 518 396467 10.1128/mr.43.4.496-518.1979 PMC281490

[B55] Lerat E Moran NA The evolutionary history of quorum-sensing systems in bacteria Mol Biol Evol 2004 21 5 903 913 10.1093/molbev/msh097 15014168

[B56] Wang X de Boer PAJ Rothfield LI A factor that positively regulates cell division by activating transcription of the major cluster of essential cell division genes of *Escherichia coli* EMBO J 1991 10 11 3363 3372 1915297 10.1002/j.1460-2075.1991.tb04900.x PMC453064

[B57] Michael B Smith JN Swift S Heffron F Ahmer BMM SdiA of *Salmonella enterica *is a LuxR homolog that detects mixed microbial communities J Bacteriol 2001 183 19 5733 5742 10.1128/JB.183.19.5733-5742.2001 11544237 PMC95466

[B58] Xavier KB Bassler BL Interference with AI-2-mediated bacterial cell-cell communication Nature 2005 437 750 753 10.1038/nature03960 16193054 PMC1388276

[B59] Winans SC Bacterial esperanto Nat Struct Biol 2002 9 2 83 84 10.1038/nsb0202-83 11813009

[B60] Winzer K Hardie KR Williams P LuxS and autoinducer-2 their contribution to quorum sensing and metabolism in bacteria Adv Appl Microbiol 2003 53 291 396 full_text 14696323 10.1016/s0065-2164(03)53009-x

[B61] Rezzonico F Duffy B Lack of genomic evidence of AI-2 receptors suggests a non-quorum sensing role for *luxS *in most bacteria BMC Microbiol 2008 8 154 10.1186/1471-2180-8-154 18803868 PMC2561040

[B62] Reading NC Sperandio V Quorum sensing the many languages of bacteria FEMS Microbiol Lett 2006 254 1 1 11 10.1111/j.1574-6968.2005.00001.x 16451172

[B63] Taga ME Miller ST Bassler BL Lsr-mediated transport and processing of AI-2 in *Salmonella typhimurium* Mol Microbiol 2003 50 4 1411 1427 10.1046/j.1365-2958.2003.03781.x 14622426

[B64] Rezzonico F Duffy B The role of *luxS *in the fire blight pathogen *Erwinia amylovora *is limited to metabolism and does not involve quorum sensing Mol Plant-Microbe Interact 2007 20 10 1284 1297 10.1094/MPMI-20-10-1284 17918630

[B65] Expert D Withholding and exchanging iron interactions between *Erwinia *spp. and their host plants Annu Rev Phytopathol 1999 37 307 334 10.1146/annurev.phyto.37.1.307 11701826

[B66] Dellagi A Brisset M-N Paulin J-P Expert D Dual role of desferrioxamine in *Erwinia amylovora *pathogenicity Mol Plant-Microbe Interact 1998 11 8 734 742 10.1094/MPMI.1998.11.8.734 9675889

[B67] Kachadourian R Dellagi A Laurent J Bricard L Kunesch G Expert D Desferrioxamine-dependent iron transport in *Erwinia amylovora *CFBP 1430 cloning of the gene encoding the ferrioxamine receptor FoxR BioMetals 1996 9 2 143 150 10.1007/BF00144619 8744897

[B68] Expert D Dellagi A Kachadourian R Vanneste JL Iron and fire blight role in pathogenicity of desferrioxamine E, the main siderophore of *Erwinia amylovora* Fire blight the disease and its causative agent, Erwinia amylovora 2000 Wallingford, UK CAB International 179 195 full_text

[B69] Feistner GJ Proferrioxamine synthesis in *Erwinia amylovora *in response to precursor or hydroxylysine feeding metabolic profiling with liquid chromatography-electrospray mass spectrometry BioMetals 1995 8 4 318 327 10.1007/BF00141605 7580052

[B70] Darling ACE Mau B Blattner FR Perna NT Mauve multiple alignment of conserved genomic sequence with rearrangements Genome Res 2004 14 1394 1403 10.1101/gr.2289704 15231754 PMC442156

[B71] Roselló M Ferrer S Llop P López MM Christen R Gardan L Description of *Erwinia piriflorinigrans *sp. nov., causal agent of pear blossom necrosis Acta Hort 2008 793 137 140

[B72] Chung YR Brenner DJ Steigerwalt AG Kim BS Kim HT Cho KY *Enterobacter pyrinus *sp. nov., an organism associated with brown leaf spot disease of pear trees Int J Syst Bacteriol 1993 43 157 161

[B73] Hao MV Brenner DJ Steigerwalt AG Kosako Y Komagata K *Erwinia persicinus *a new species isolated from plants Int J Syst Bacteriol 1990 40 379 383 2275853 10.1099/00207713-40-4-379

[B74] Stockwell VO Hockett K Marie C Duffy B Pink *Erwinia amylovora *colony discoloration in diagnostic isolations by co-cultured bacteria Acta Hort 2008 793 539 542

[B75] Jock S Geider K Molecular differentiation of *Erwinia amylovora *strains from North America and of two Asian pear pathogens by analyses of PFGE patterns and *hrpN *genes Environ Microbiol 2004 6 5 480 490 10.1111/j.1462-2920.2004.00583.x 15049921

[B76] Geider K Auling G Du Z Jakovljevic V Jock S Völksch B *Erwinia tasmaniensis *sp. nov., a non-phytopathogenic bacterium from apple and pear trees Int J Syst Evol Microbiol 2006 56 12 2937 2943 10.1099/ijs.0.64032-0 17159002

[B77] Mergaert J Hauben L Cnockaert MC Swings J Reclassification of non-pigmented *Erwinia herbicola *strains from trees as *Erwinia billingiae *sp. nov Int J Syst Bacteriol 1999 49 377 383 10319458 10.1099/00207713-49-2-377

[B78] Hernandez d François P Farinelli L Østerås M Schrenzel J De novo bacterial genome sequencing millions of very short reads assembled on a desktop computer Genome Res 2008 18 802 809 10.1101/gr.072033.107 18332092 PMC2336802

[B79] McHardy AC Goesmann A Pühler A Meyer F Development of joint application strategies for two microbial gene finders Bioinformatics 2004 20 10 1622 1631 10.1093/bioinformatics/bth137 14988122

[B80] Salzberg SL Delcher AL Kasif S White O Microbial gene identification using interpolated Markov models Nucleic Acids Res 1998 26 2 544 548 10.1093/nar/26.2.544 9421513 PMC147303

[B81] Badger JH Olsen GJ CRITICA coding region identification tool invoking comparative analysis Mol Biol Evol 1999 16 4 512 524 10331277 10.1093/oxfordjournals.molbev.a026133

[B82] Kanehisa M Goto S Kawashima S Nakaya A The KEGG databases at GenomeNet Nucleic Acids Res 2002 30 1 42 46 10.1093/nar/30.1.42 11752249 PMC99091

